# Polydextrose Addition Improves the Chewiness and Extended Shelf-Life of Chinese Steamed Bread Through the Formation of a Sticky, Elastic Network Structure

**DOI:** 10.3390/gels11070545

**Published:** 2025-07-14

**Authors:** Chang Liu, Bing Dai, Xiaohong Luo, Hongdong Song, Xingjun Li

**Affiliations:** 1 Academy of National Food and Strategic Reserves Administration, National Engineering Research Center for Grain Storage and Transportation, Beijing 102209, China; shlglc@126.com (C.L.); bingdai3@126.com (B.D.); lxh@ags.ac.cn (X.L.); 2 College of Health Science and Engineering, University of Shanghai for Science and Technology, Shanghai 200093, China; cau4080@163.com; 3 College of Grain and Strategic Reserves, Henan University of Technology, Zhengzhou 450001, China

**Keywords:** polydextrose, Chinese steamed bread, continuous three-dimensional network structure, freshness and looseness, dough development time, water soluble dietary fiber

## Abstract

This study explored the effects of adding a newly developed type of polydextrose on the appearance, sensory score, and textural parameters of steamed bread and the microstructure of dough, as well as the pasting, thermal, and thermal mechanical properties of high-gluten wheat flours. The results revealed that, compared with a control sample, 3–10% of polydextrose addition significantly increased the hardness, adhesiveness, gumminess, and chewiness of steamed bread, but other textural parameters like springiness, cohesiveness, and resilience remained basically the same. Further, in contrast to the control sample, 3–10% polydextrose addition significantly reduced the specific volume and width/height ratio of steamed bread but increased the brightness index, yellowish color, and color difference; improved the internal structure; and maintained the other sensory parameters and total score. Polydextrose addition decreased the peak, trough, final, breakdown, and setback viscosity of the pasting of wheat flour suspension solutions but increased the pasting temperature. Polydextrose additions significantly reduced the enthalpy of gelatinization and the aging rate of flour paste but increased the peak temperature of gelatinization. A Mixolab revealed that, with increases in the amount of added polydextrose, the dough’s development time and heating rate increased, but the proteins weakened, and the peak torque of gelatinization, starch breakdown, and starch setback torque all decreased. Polydextrose additions increased the crystalline regions of starch, the interaction between proteins and starch, and the β-sheet percentage of wheat dough without yeast and of steamed bread. The amorphous regions of starch were increased in dough through adding polydextrose, but they were decreased in steamed bread. Further, 3–10%of polydextrose addition decreased the random coils, α-helixes, and β-turns in dough, but the 3–7% polydextrose addition maintained or increased these conformations in steamed bread, while 10% polydextrose decreased them. In unfermented dough, as a hydrogel, the 5–7% polydextrose addition resulted in the formation of a continuous three-dimensional network structure with certain adhesiveness and elasticity, with increases in the porosity and gas-holding capacity of the product. Moreover, the 10% polydextrose addition further increased the viscosity, freshness, and looseness of the dough, with smaller and more numerous holes and indistinct boundaries between starch granules. These results indicate that the 3–10% polydextrose addition increases the chewiness and freshness of steamed bread by improving the gluten network structure. This study will promote the addition of polydextrose in steamed bread to improve shelf-life and dietary fiber contents.

## 1. Introduction

A report on the nutrition and chronic disease status of Chinese residents indicated that in 2014, the number of overweight and obese people in China reached 225 million and 125 million, respectively, while there were 160 million hypertensive patients, 160 million people with lipid disorder, and 94 million individuals with abnormal blood sugar (diabetes) [1,2]. In 2019, the premature mortality rate among patients with major chronic diseases, including cardiovascular and cerebrovascular diseases, cancer, chronic respiratory diseases, and diabetes, in China was 16.5%, 2% lower than in 2015 [3]. The Report on the Monitoring of Chronic Diseases in the National Plan, published in March 2024, indicated that there were more than 480 million chronic patients in China in 2023, accounting for 30.5% of the total population [4]. The main cause is a lack of dietary fiber intake by residents [5]. Fiber is the indigestible component of plants. It does not provide any nutrients to the body, but it is essential for maintaining the healthy function of the human digestive system [6,7]. Cereal dietary fibers can prevent and alleviate diabetes, plasma cholesterol, cardiovascular diseases, rectal cancer, emphysema, and obesity [8,9]. Whole wheat steamed bread, with a dietary fiber content of 10%, has a very poor taste, but delicious white steamed bread only has about 1%dietary fiber [10,11]. White steamed bread is a staple traditional fermented food in China and Asia that is consumed by a large proportion of residents [12,13]. Thus, it is worth studying methods to increase the dietary fiber in steamed white bread through the addition of water-soluble dietary fibers.

The physical properties of fiber-containing foods allow them to regulate the colon, in part because of their ability to bind to water [14]. Polydextrose has a strong water-holding capacity [15]. Polydextrose is a highly branched polymer with randomly distributed α- and β-glycosidic bonds. It is synthesized with D-glucose in a reactor at a ratio of 89:10:1 (D-glucose–sorbitol–citric acid) [16]. Polydextrose has been used in the food industry in developed countries as a bulking agent and a low-energy ingredient, replacing sugar and fat since 1980 [17,18]. China introduced and synthesized this product in the year 2000. China produced 101,000 tons of polydextrose in 2020, with an annual output value of around USD 0.137 billion; production in 2026 is expected to reach 135,000 tons [19]. The use of polydextrose in health foods, beverages, fermented dairy products, and baked foods accounts for 39%, 21.6%, 16.6%, and 11.8%, respectively, of the total use of polydextrose in China, and the remaining 11% is used in chocolate, nutrition bars, and other foods [19]. Polydextrose is good for human health as a soluble dietary fiber [20]. Compared with insoluble dietary fiber, polydextrose has many more health and processing benefits. Polydextrose can be widely utilized in various foods, including high-fiber, low-energy functional foods, because it has fewer calories (1 cal/g), better stability, and high tolerance to sugars [21]. Polydextrose can also be used as a humectant in baked foods; it can delay moisture evaporation, prevent the product from spoiling, and extend shelf-life [22]. Polydextrose has excellent water-binding properties that help stabilize gluten [23] or bread dough [24] during freezing and thawing processes. Jing et al. [10] reported that a 1–9% addition of polydextrose as a soluble fiber had little effect on the sensory attributes of steamed bread. However, there is a lack of further research on the addition of polydextrose to steamed bread during dough preparation and the resulting effects on the freshness of the cooked steamed bread.

Steamed bread, made by steaming fermented dough, has a history of more than 1700 years. It is a popular food in China and many Asian countries [25]. However, there are differences in the dietary structure of steamed bread compared to whole wheat flour itself, and steamed bread contains low levels of lysine, vitamins, minerals, and dietary fiber; its nutritional components are not comprehensive nor balanced [25,26]. In this study, given the high moisture absorption rate of amorphous polydextrose powder, a new type of polydextrose (PD-ST) with high market cost performance was used, and 3–10% this polydextrose was added as the soluble fiber during the dough preparation of white steamed bread; the appearance, tasting score, and textural parameters of the steamed bread, as well as its gelatinization parameters and thermo-mechanical properties, were analyzed. The crystal conformation of starch and the secondary structure of protein in dough and steamed bread were comprehensively analyzed, and the microstructure of the unfermented dough was observed. The aim of this study was to improve the shelf-life, chewiness quality, and dietary fiber content of fermented steamed bread through the addition of polydextrose, thereby preventing and alleviating the incidence of chronic diseases in residents who mainly eat steamed bread.

## 2. Results and Discussion

### 2.1. Comparison of New Polydextrose and Conventional Polydextrose

**Figure 1:**
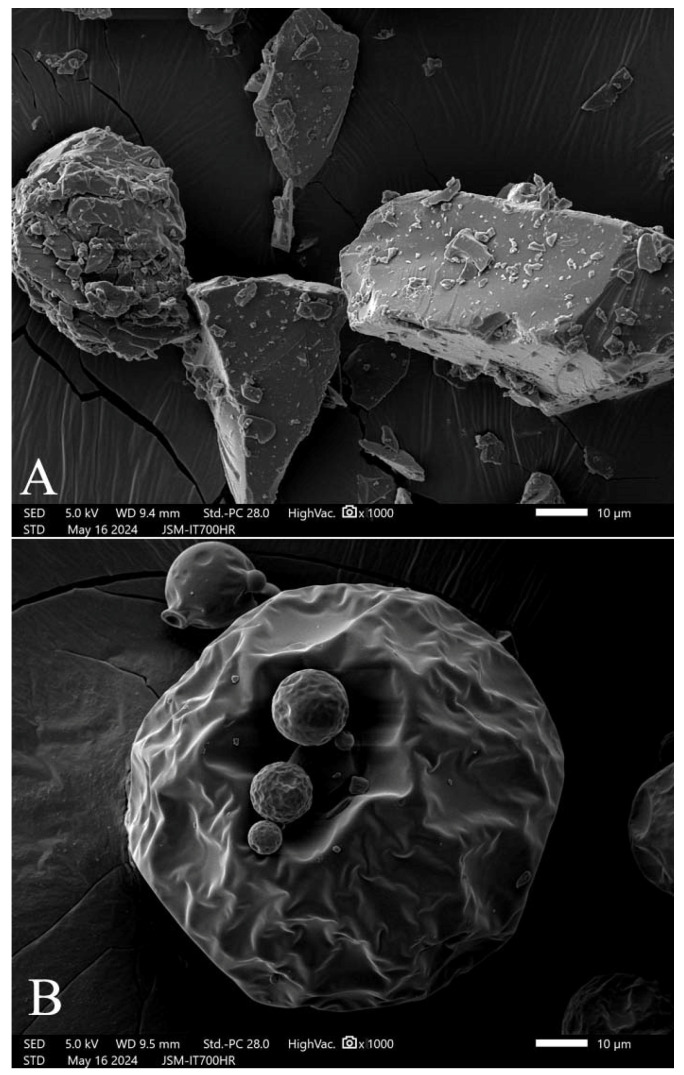
New polydextrose (PD-ST) and conventional polydextrose (PD-XG). Note: (**A**) PD-ST; (**B**) PD-XG. The photos are enlarged by1000×.

**Figure 2:**
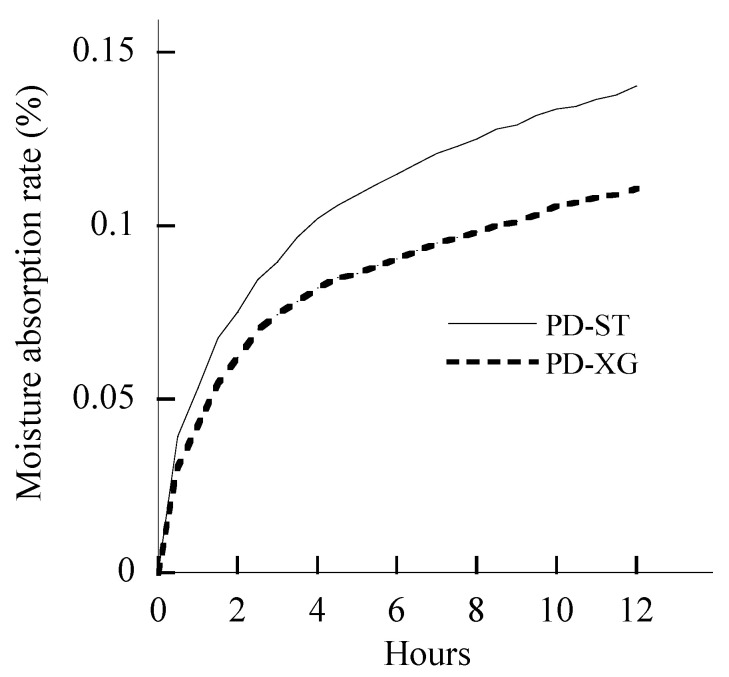
The moisture absorption rate of PD-ST and PD-XG polydextrose. Note: The data are expressed as mean; number of repetitions—*n* = 2.

New polydextrose (PD-ST), with 1.0% moisture content (MC), consists of irregular and inhomogeneous particles (Figure 1A), and it is different from conventional spherical polydextrose with protrusions (PD-XG, 4.7% MC) (Figure 1B). PD-ST has a higher moisture absorption rate than PD-XG over a 12 h measurement period at 26 °C and 84.1% relative humidity (Figure 2).

**Figure 3:**
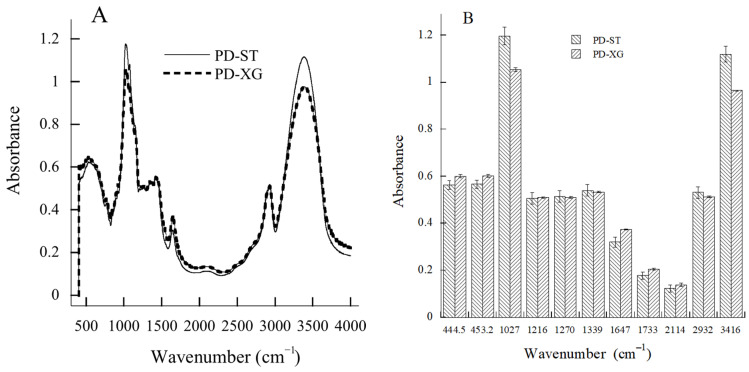
The absorbance of characteristic peaks in the FTIR of PD-ST and PD-XG polydextrose. Note: (**A**) IR scanning curves; (**B**) Characteristic peaks; Number of repetitions—*n* = 3.

Two polydextrose samples had at least 11 infrared spectroscopy absorption peaks (Figure 3A), and the peaks with an absorbance value of greater than 1 were 1027 and 3416 cm^−1^, representing amorphous structure (primary alcohol C-OH) and hydroxyl functional groups, respectively (Figure 3B). This is a significant feature of amorphous-phase polydextrose powder. The absorption value of these two functional groups in PD-ST is significantly greater than that of PD-XG. The peaks at 1216 and 1270 cm^−1^ correspond to the stretching vibration peaks of the C-OH and CH_2_OH groups, respectively, and the 1339, 1647, and 2932 cm^−1^ peaks are the CH and CH_2_ groups [27]. The peak at 1733 cm^−1^ corresponds to the C=O group [27].

Both types of polydextrose have similar purity levels and contents of small molecular sugars, complying with GB1986.385-2025 [28]. Compared with the moisture content (4.66 ± 0.03% wet basis) of PD-XG polydextrose, PD-ST shows a significant decrease in moisture content (1.00% ± 0.01%) and has high performance costs. The important process feature of PD-ST is that it was vacuum-dried during the entire polymerization reaction cycle in the reactor. Polydextrose is a versatile ingredient in the food industry, possibly due to its amorphous powder state (Figure 1) and strong water-holding capacity (Figure 2). The present study used PD-ST to carry out ana dditive test for wheat dough. The observed stronger hydroxyl peaks in PD-ST might be linked to increased hydrophilicity or a greater potential for hydrogen bonding with flour components.

### 2.2. Effect of Added Polydextrose on the Texture Profile and Appearance of Steamed Bread

Compared with the control sample, the 3–10% polydextrose PD-ST addition significantly increased the adhesiveness, hardness, gumminess, and chewiness of steamed bread, but the resilience, cohesiveness, and springiness basically remained the same, and the adhesive force was reduced (Table 1).

The 3–10% polydextrose addition decreased the specific volume of steamed bread but increased its mass (Table 2). With respect to the steamed bread made from high-gluten wheat flour, the present study showed that polydextrose PD-ST increased their volumes at 7% and 10% addition and increased mass at 3–10% addition. These results are essentially similar to the results of Jing [10], where 1%, 5%, 7%, and 9% polydextrose additions significantly increased the volume and specific volume of steamed bread and 3%, 7%, and 9% polydextrose additions increased the mass of steamed bread (the steamed bread might be processed from medium-gluten flour). Our results suggest that, with an increase in polydextrose addition, raw steamed bread with the same weight will absorb more steam from the steamer during the steaming process, resulting in an increase in both the volume and weight of the steamed bread. The steamed bread containing 3–10% PD-ST had similar specific volumes, and this is possibly due to their similar volumes.

The brightness index, yellowish color, and color difference of steamed bread were significantly increased by the 5–10% polydextrose addition (Table 3). Compared with the control sample, 3%, 5%, 7%, and 10% polydextrose additions increased the color difference by 5.8%, 15.8%, 14.6%, and 16.0%, respectively.

### 2.3. Effect of Added Polydextrose on the Sensory Attributes of Steamed Bread

**Figure 4:**
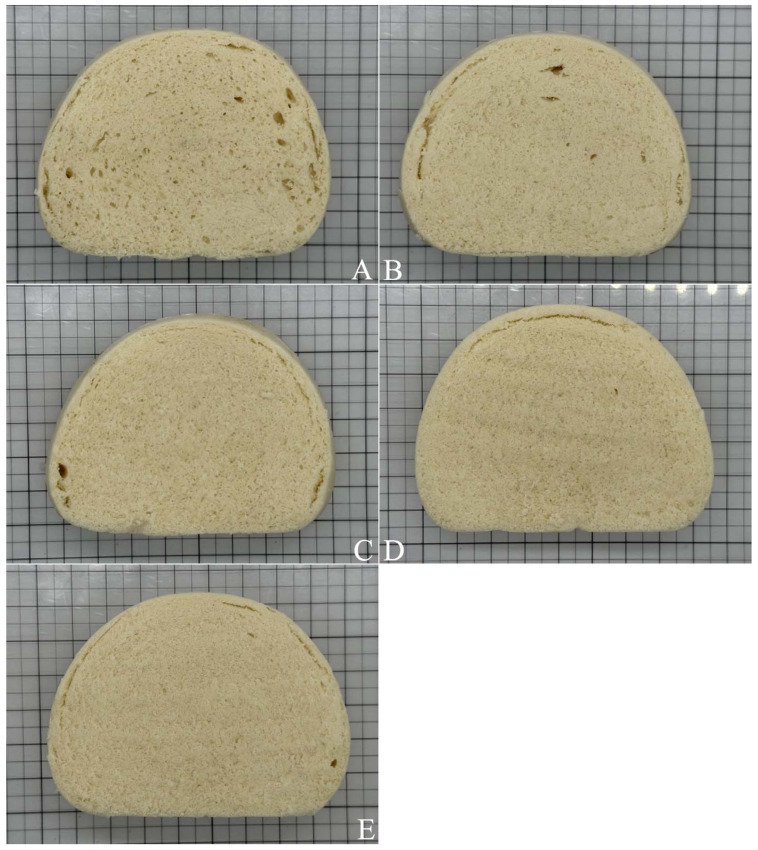
Cross-section of steamed bread. Note: (**A**) is the control without polydextrose; (**B**–**E**) are 3%, 5%, 7%, and 10% PD additions. All photos are enlarged 1×.

Table 4 shows that the 3–10% polydextrose addition significantly reduced the specific volume and width/height ratio of the steamed bread; improved its internal structure; and maintained the elasticity, surface color and structure, toughness, adhesiveness, taste, and total score. The panel for the sensory analysis was trained by the experienced experts. The cross-sections in Figure 4 show that the steamed bread became uniform in texture, with fewer holes appearing as the amount of polydextrose added increased.

### 2.4. Effect of Added Polydextrose on the Pasting and Thermal Parameters of Wheat Flours

Table 5 shows that polydextrose additions decreased the peak, trough, and final viscosity, as well as the breakdown and setback viscosity of the pasting of wheat flour suspension solutions, but increased the pasting temperature; the gelatinization peak time tended to reduce.

Table 6 shows that when determining the thermal properties of flour paste at 0 d, compared with the control sample, the addition of polydextrose significantly reduced the enthalpy value and peak width of gelatinization but increased the onset temperature, peak temperature, and conclusion temperature of gelatinization; the peak width of gelatinization essentially remained the same. The PD-ST addition increased the peak temperature of gelatinization in wheat flours, which is similar to the results of Rosenthal [29], where polydextrose elevated the starch gelatinization temperature. Our study suggests that polydextrose, with its high water-binding capacity, could compete with starch for water molecules during gelatinization, leading to reduced peak viscosity and gelatinization enthalpy.

Table 7 further shows that when the retrograded samples were kept at 4 °C for 21 days and the thermal properties were then re-measured, compared with the control sample, the addition of polydextrose significantly reduced the enthalpy value, peak width, and peak width of gelatinization but increased the onset temperature; the peak temperature of gelatinization remained unchanged. The aging rate of wheat flour paste was significantly reduced by the addition of polydextrose.

### 2.5. Effect of Added Polydextrose on the Thermo-Mechanical Property of Wheat Dough Without Yeast Powder

Table 8 shows that with an increase in the amount of added polydextrose, the dough’s development time significantly increased, and the dough stability time first increased and then decreased, reaching a maximum at 5% polydextrose addition. The protein’s weakening degree, peak torque of gelatinization, starch breakdown, and starch setback torque all increased while α-amylase and enzymatic hydrolysis rates remained unchanged. The heating rate increased, and the gelatinization rate first increased and then decreased. The 3–10% PD-ST addition decreased the dough’s protein weakness and starch gelatinization torque, but the dough’s gelatinization speed was retained or even increased by the 3–7% PD-ST addition, and it decreased via the10% PD-ST addition, suggesting that PD-ST additions could reinforce the gluten matrix, increase pasting temperatures, and decrease the viscosity of flour through hydration effects.

### 2.6. Effect of Added Polydextrose on Starch Crystallinity and Protein Conformation

FTIR analysis showed that the R_1022/995_ of unfermented dough was significantly greater than that of the steamed bread, but its R_1047/1022_ and R_1068/1022_ ratios were significantly lower than those of steamed bread (Table 9 and Table 10). Moreover, the percentage of β-sheets in unfermented dough was significantly lower than that in steamed bread, but its contents of random coils, α-helixes, and β-turns were significantly higher. These results suggest that, in the presence of a proper amount of water molecules, the higher amorphous regions of starch in the dough, as well as the higher relative contents of secondary structures like random coils, α-helixes, and β-turns, resulted in the orderly state of the gluten network structure.

Compared with the control sample, the addition of polydextrose increased the amorphous and crystalline regions of starch and the interaction between proteins and starch in wheat dough without yeast (Table 9). The percentage of β-sheets accounted for around 50% of the secondary protein structure in the wheat dough. The 3–7% polydextrose addition significantly increased the β-sheets but decreased the random coils, α-helixes, and β-turns.

For steamed bread, the addition of polydextrose decreased the amorphous regions of starch but increased the crystalline regions of starch and the interaction between protein and starch (Table 10). Compared with the dough, the β-sheet conformation in steamed bread was increased by 2%, but random coils, α-helixes, and β-turns were reduced. The 3–7% polydextrose addition significantly increased the amount of β-sheets and the percentages of random coils, α-helixes, and β-turns in steamed bread, but the 10% polydextrose addition decreased the conformations of random coils, α-helixes, and β-turns. These results of moderate PD-ST (3–7%) could promote ordered secondary protein structures, while high PD-ST (10%) might disrupt protein folding, possibly due to excessive water competition or steric hindrance.

### 2.7. Effect of Added Polydextrose on the Microstructure of Unfermented Dough

**Figure 5:**
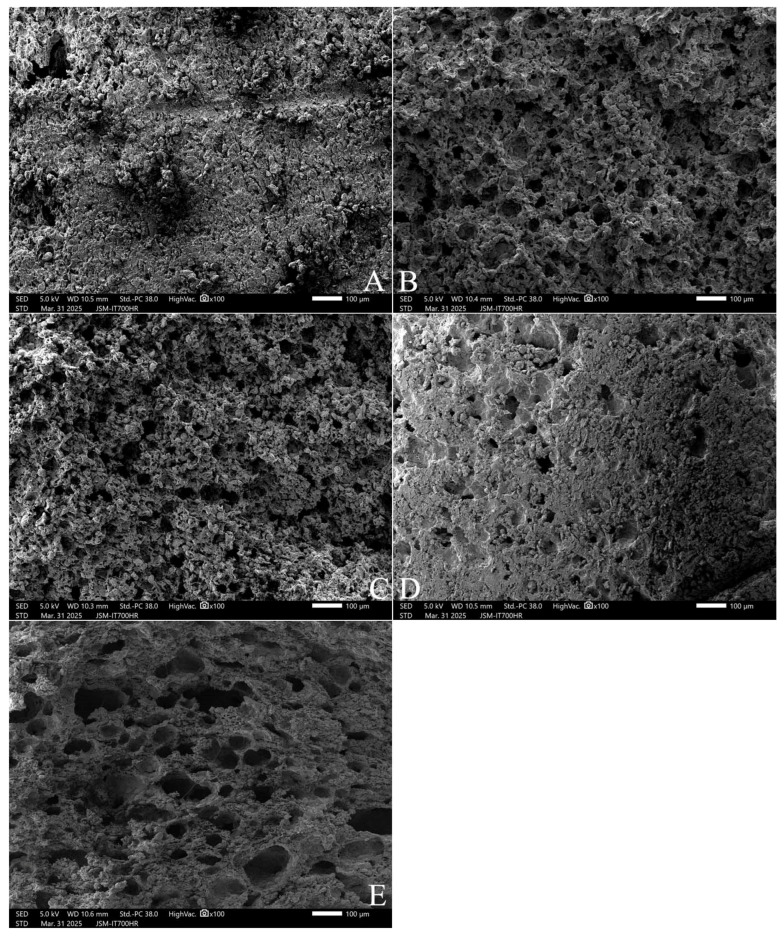
Microstructure of the freeze-dried cubes of unfermented dough (100×).Note: (**A**) is the control without polydextrose; (**B**–**E**) are 3%, 5%, 7%, and 10% PD-ST additions. All photos are enlarged 100×.

When the freeze-dried cubes of unfermented dough were observed at an enlargement of 100 times, the surface of the dough was uneven, and it occasionally had larger and deeper holes with irregular openings (Figure 5A). With an increase in the addition concentrations of polydextrose, the holes on the surface of the dough increased significantly (Figure 5B,C), the openings were approximately circular, and the holes gradually deepened (Figure 5D). In particular, with the 10% PD-ST addition, the number of holes increased, holes with larger openings increased, and the holes became deeper (Figure 5E). These results are basically similar to the results shown in Figure 4. These results suggest that the hydroxyl groups in strongly hydrophilic polydextrose molecules might combine with the carboxyl groups of gluten proteins, resulting in an even distribution of polydextrose molecules in the gluten network. When the samples for electron microscope observation were prepared by the freeze-drying method, the water molecules escaped, leaving holes with relatively uniform diameters in the gluten network. These conclusions can be further confirmed in wheat flour dough using laser confocal scanning microscopy (CLSM) or quantified with image analysis software.

**Figure 6:**
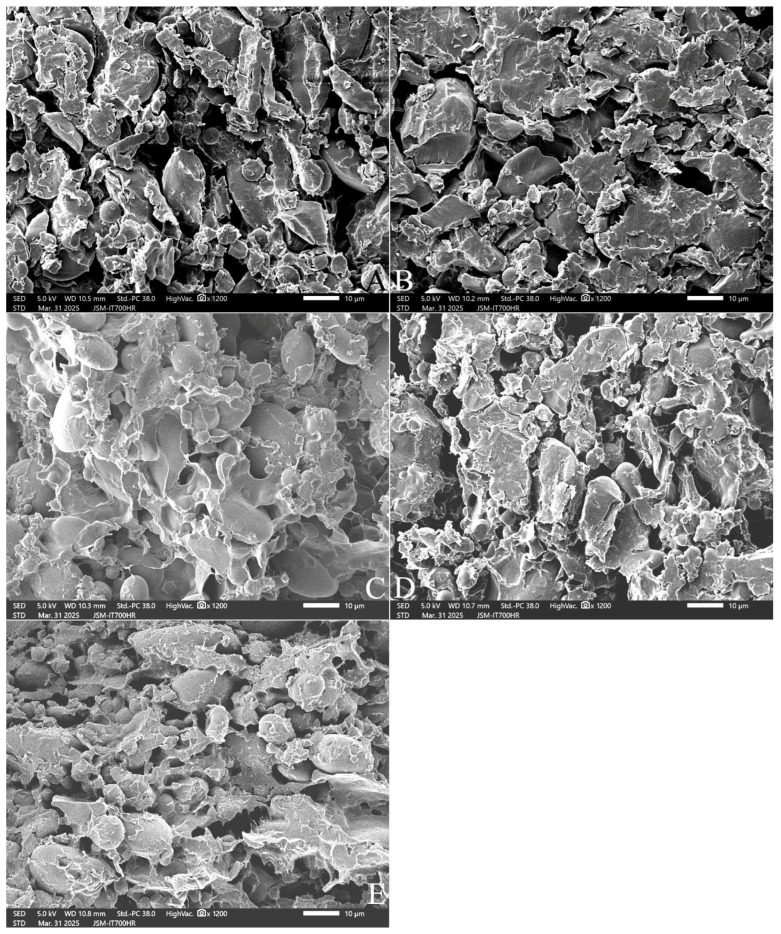
Microstructure of the freeze-dried cubes of unfermented dough (1200×).Note: (**A**) is the control without polydextrose; (**B**–**E**) are 3%, 5%, 7%, and 10% PD-ST additions. All photos are enlarged to 1200×.

Figure 6 further shows the microstructure of the dough at an enlargement of 1200 times. In the dough without added polydextrose, spherical or ellipsoidal starch granules were exposed on the surface, in the interstices of the solidified gluten protein network, or embedded in the gluten protein network, with clear gaps between the starch granules and clear boundaries (Figure 6A). Polydextrose relies on its unique structure to form a continuous three-dimensional network structure with certain adhesion and elasticity, with the 3–7% polydextrose addition playing a similar role to the gluten network structure, gradually increasing the uniform pores and even the bubble-holding capacity of the dough (Figure 6B–D). At 10% polydextrose addition, the adhesion, freshness, and looseness of the dough were further increased, the holes became smaller and more numerous, and the boundaries between starch granules became blurred (Figure 6E).

Dietary fiber is more widely used in baked foods in Western countries [7]. The amount of added dietary fiber is generally 5–10% of the flour content. If the amount exceeds 10%, it will slow down the fermentation speed of the bread [30,31]. In the present study, the 3–10% polydextrose (PD-ST) addition was tested in steamed breads made from high-gluten wheat flours. These addition levels maintained most of the sensory evaluation parameters of the steamed bread, such as its elasticity, surface color and structure, toughness, adhesiveness, taste, and total score, despite significantly reducing the specific volume and width/height ratio of the bread. These results are similar to the results of Jing et al. [10], where the 1–9% polydextrose addition had little effect on the sensory evaluation of steamed bread (their bread may be made from middle-gluten wheat flours). Our result is different from that of a previous study where the addition of konjac glucomannan was found to reduce the hardness of steamed bread [32], possibly due to the hygroscopic properties of different polysaccharides [33]. The increase in the brightness, color difference, hardness, adhesiveness, gumminess, and chewiness of steamed bread by the addition of 5–10% polydextrose could be explained by the three-dimensional network structure of the unfermented dough with certain adhesion and freshness properties, as observed under SEM.

Polydextrose has been incorporated into a wide range of foods, including baked goods, beverages, confectionery, and frozen desserts [17]; however, few studies have meticulously investigated the effects of polydextrose addition in steamed bread. Among 1–9% polydextrose additions, Jing et al. [10] reported that a 3% polydextrose addition improved the maximal hardness and chewiness, while other addition amounts resulted in no changes relative to the control sample. In the present study, the 3–10% polydextrose addition to steamed bread resulted in similar textural parameters, like springiness, cohesiveness, and resilience, to the control sample, except for the increase in hardness, adhesiveness, gumminess, and chewiness and a decreasing trend in adhesive force. Schirmer et al. [18] added 0–21.96% polydextrose into the batter of pound cake as a replacement for sucrose and found that the firmness of the batter was unchanged, but its adhesive force significantly increased. Further, the firmness and springiness of the pound cake remained unchanged with 0–17.57% polydextrose additions, but they were significantly reduced with a 21.96% polydextrose addition. Additionally, 0–21.96% polydextrose additions significantly increased the specific volume of the cake. These results suggest that 0–10% polydextrose additions to steamed bread or baked pound cake retain the cohesiveness and springiness of the product. Further studies should explore the effects of 20% polydextrose additions on the textural parameters of steamed bread.

Our recent study showed that the 3–10% polydextrose addition significantly decreased the hardness, adhesive force, adhesiveness, cohesiveness, gumminess, and chewiness of cooked Indica rice [34]. In the present study, the 3–10% polydextrose addition to steamed bread resulted in an increase in hardness, adhesiveness, gumminess and chewiness but retained similar textural parameters, like springiness, cohesiveness, and resilience, as the control sample, suggesting that polydextrose might play different roles in the interaction between starch and protein in cooked rice versus steamed bread through intermolecular interactions and the formation of hydrogel-like networks.

Early analyses of the microstructure of cake batters with polydextrose substitutions showed that the addition of 14% and 28.1% polydextrose in cake batters resulted in a better air-holding capacity, which resulted in an increase in bubble size [35], but Hicsasmaz et al. [36] showed that an increase in the polydextrose content from 0% to 28.1% led to a decrease in the number and size of crack-like pores, with an increase in bubble count in the cake batter. Kocer et al. [37] added 0–28.1% polydextrose to replace sugar in a high-ratio cake and found that the uniformity of the pore sizes in the crumb increased with increasing amounts of added polydextrose. In the present study, a 10% polydextrose addition resulted in significantly more sphere-like pores than crack-like pores in the unfermented dough cube, along with increased uniformity of pore sizes. The adhesion, freshness, and looseness of the dough increased with the amount of polydextrose added. We conclude that with an increase in polydextrose addition, raw steamed bread of the same weight will absorb more steam from a steamer during the steaming process, resulting in an increase in both the volume and weight of steamed bread, as well as improved freshness and looseness.

In the present study, the relative percentage of β-sheets in unfermented dough was significantly lower than that in steamed bread, but the α-helix and β-turn percentages were significantly higher than those in steamed bread, which is consistent with the report of Ding et al. [38].The content of random coils in the unfermented dough in our study was higher than that in the steamed bread. This result is different from that of Ding et al. [38], possibly because we used infrared spectroscopy in the range of 1670–1600 cm^−1^ to analyze the secondary structure of protein, while Ding et al. [38] used infrared spectroscopy in the range of 1330–1220 cm^−1^ to analyze the secondary structure of proteins [39].

Polydextrose additions can prevent baked products from spoiling and extend their shelf life [22]. In the present study, the 3–10% polydextrose addition significantly decreased the setback viscosity of high-gluten wheat flour and the aging of retrograded flour paste, and 5–10% polydextrose reduced the starch setback torque (C5–C4) of dough, suggesting that polydextrose additions can prevent steamed bread from aging during storage and maintain its springiness and cohesiveness. Further studies are needed to define the effect of polydextrose (PD-ST) on the textural profile of steamed bread made from middle- or low-gluten wheat flours, as well as on the dough microstructure, pasting, thermal, and thermal mechanical properties, and to determine the best amount of polydextrose addition.

## 3. Conclusions

The addition of 3–10% polydextrose (PD-ST) to high-gluten wheat flour modified its rheological, thermal, and thermo-mechanical properties. It decreased the peak, breakdown, and setback viscosity of pasting; reduced the enthalpy of gelatinization and the aging rate of flour paste while increasing the peak temperature of gelatinization; and decreased the protein weakening degree, peak torque of gelatinization, starch breakdown, and starch setback torque while increasing dough development time.

The addition of polydextrose increased the crystalline regions of starch, the interaction between protein and starch, and the β-sheet percentage of wheat dough and steamed bread. The addition of polydextrose also increased the amorphous regions of starch in the dough but decreased these in steamed bread. Further, the 3–10% polydextrose addition decreased the percentages of random coils, α-helixes, and β-turns in the dough, but the 3–7% polydextrose addition maintained or increased these conformations in steamed bread, while 10% polydextrose decreased them.

The 3–10% polydextrose addition decreased the specific volume and width/height ratio of the steamed bread but increased the brightness index, hardness, adhesiveness, gumminess, and chewiness. The microstructure results indicated that the 3–10% polydextrose addition resulted in the formation of a continuous three-dimensional network structure with certain adhesion and elasticity in the unfermented dough, increasing the number of small pores and the gas-holding capacity of the product through unique molecular interactions. Polydextrose could alter dough rheology and the texture of steamed bread by competing with starch for water and interfering with gluten formation.

In summary, the addition of 3–10% polydextrose to steamed bread not only increased the dietary fiber content of the product but also improved its chewiness and shelf life.

## 4. Materials and Methods

### 4.1. Samples

**Figure 7:**
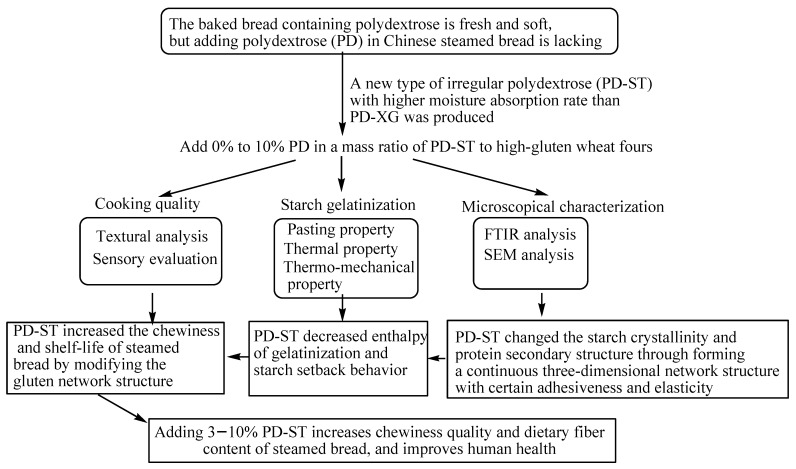
A schematic diagram of the present study.

Polydextrose ST (PD-ST) with an initial moisture content of 1.0% was provided by Runloy Biotechnology (Shanghai) Co., Ltd., Shanghai, China. Polydextrose XG (PD-XG) was provided by Xingguang Co., Ltd., Binzhou, China. High-gluten wheat core flour (Golden Dragonfish, Wilmar Shanghai Co., Ltd., Shanghai, China) was purchased from a local supermarket. PD-ST was added to wheat flour at mass ratios of 0%, 3%, 5%, 7%, and 10%, respectively, to obtain polydextrose-added samples. The measured analysis parameters and important experimental steps are shown in Figure 7.

### 4.2. The Moisture Absorption Rate of Polydextrose

The method for determining the moisture absorption of the sample is as follows: 1 g of polydextrose was weighed in an aluminum box (diameter of 5 cm and height of 3 cm) and placed on a steel wire rack in the middle of an incubator (26 °C). A ceramic plate filled with distilled water was placed at the bottom of the insulated oven incubator (DHG 9070A, Blue Sky Chemical Instrument Factory, Hangzhou, China), and the weight of the aluminum box containing the sample was weighed every half an hour for 12 h. The relative humidity was 84.1%, which was measured by a hygrometer.

### 4.3. Textural Profile of Wheat Steamed Bread

The textural profile of wheat steamed bread was analyzed with a textural analyzer (CTX, Brookfield, Middleboro, MA, USA), as described by Wang et al. [34]. Wheat steamed bread was made according to GB/T 35991-2018 [40]. A flour sample (200 g) was weighed and mixed with 40 g of warm water (38 °C) containing 1.6 g of yeast. The dough was then kneaded for 180 s in a kneading machine at room temperature after the addition of 50 g of deionized water. Subsequently, the dough was taken out and pressed into films 6–8 times using a rolling machine to move the bubbles. It was then divided into three equal parts. Subsequently, the dough was kneaded and folded towards the bottom edge to ensure that the front and back of the dough were kneaded the same number of times. The well-kneaded dough was fermented for 30 min at 35 °C and 85% relative humidity in a fermentation box. Then, 1500 mL of deionized water was added toa pot, and the bread was steamed for 25 min at 1600 W. It was then braised for 5 min without uncovering the pot’s lid. The steamed bread was then removed and covered with gauze to cool at room temperature for 1 h before the later determination of textural profiles.

### 4.4. Specific Volume and Color of Steamed Bread

The specific volume of steamed bread was determined according to GB/T 21118-2007 [41].The mass of the steamed bread was measured after cooling for 1 h. The volume of the steamed bread was measured by the rapeseed replacement method; the average value was taken after three repetitions. The specific volume was calculated according to the following formula:

(1) $$\mathrm{Specific}\;\mathrm{volume}\;\left(\frac{{\mathrm{cm}}^{3}}{g}\right)=\frac{\mathrm{Volume}\;\mathrm{of}\;a\;\mathrm{steamed}\;\mathrm{bread}\;\left({\mathrm{cm}}^{3}\right)}{\mathrm{Mass}\;\mathrm{of}\;a\;\mathrm{steamed}\;\mathrm{bread}\;\left(g\right)}$$

The color of steamed bread was determined using a colorimeter (CR-100, Japanese Konica Minolta, Ltd., Shanghai, China). The steamed bread was cut into slices with a thickness of 15 mm using a slicer to obtain test samples. A standard white plate was used for calibration. Then, steamed bread slices were placed on the test port, completely covering it, and the test key was pressed. The sample was removed after the test was completed to obtain a set of color differences. The colorimeter measured the brightness (L*), redness-greenness (a*), and yellowness-blueness (b*) values using the color difference between the standard white plate and the tested steamed bread slices. ΔE is the overall color difference.

The slices of steamed bread were photographed using a mobile phone (Apple, iPhone 15, 12 million pixels, Apple official flagship store, Beijing, China).

### 4.5. Pasting Characteristics

An RVA—TecMaster (PertenRuihua Scientific Instrument Co., Ltd., Beijing, China) was used to determine the pasting parameters of the samples according to the GB/T24852-2010 method [42]. During measurement, the stirring paddle speed was set at 960 r/min for the initial 10 s and then lowered to 160 r/min within 20 s and kept at 180 r/min. The temperature was initially set at 50 °C for 1 min and then raised to 95 °C within 3.71 min and kept there for 2.5 min before being reduced to 50 °C within 2.8 min and retained for 2 min.

### 4.6. Thermal Properties

Polydextrose was uniformly mixed with wheat flour on amass basis to achieve addition levels of 0% to 10%. A 5.0 mg sample was employed to determine the gelatinization parameters using a differential scanning calorimeter (DSC 214, NetzschGmbH, Selb, Germany), according to the method of Wang et al. [34]. After gelatinization, the retrograded samples were placed in 24-well plates with covers at 4 °C for 21 days and again measured for retrogradation. The results were evaluated using the same analysis software. The aging of the retrograded wheat flour paste was calculated using Equation (2):

Ageing (%) = (Gelatinization enthalpy measured at day 21)/(Gelatinization enthalpy measured at day 0) × 100(2)

### 4.7. Thermo-Mechanical Characteristics

A Mixolab (Chopin Technologies, Tripette et Renaud, Paris, France) was employed to analyze the thermo-mechanical characteristics of the dough, as described by Wang et al. [34]. For assays at constant hydration, about 50 g of wheat flour was placed into the Mixolab bowl. Then, 75 g of dough with 60% water content (14% moisture basis) was evaluated.

### 4.8. Fourier Transform Infrared Spectroscopy (FTIR)

The FTIR spectra of the cooked samples were recorded on a Nicolet 6700 FTIR spectrometer (Thermo Fisher Scientific, Greater Mumbai, MA, USA). The test conditions were as follows: spectral resolution of 4 cm^−1^ with 100% T-line signal-to-noise ratio from 4300 to 4400 cm^−1^, 64 scans. All measurements were performed with OMNIC software. Samples were ground with potassium bromide at a mass ratio of 100:1 and pressed into tablets. The scanning wavelength was 400–4000 cm^−1^. The wavelength ratios R_1022/995_ and R_1047/1022_ indicate the short-range of the starch granule’s surface, representing the amorphous and crystalline regions of starch, respectively. R_1068/1022_ represents the interaction between protein and starch [43]. The secondary structures of proteins and internal hydrogen bonding were analyzed mainly through the spectral changes in the amide I region (1700 to 1600 cm^−1^). The main protein structure forms in the amide I region are as follows: β-sheets (1600 to 1640 cm^−1^), random coils (1640 to 1650 cm^−1^), α-helixes (1650 to 1660 cm^−1^), and β-turns (1660 to 1700 cm^−1^). The changes in these four structures can reflect the changes in the secondary structures of proteins [44].

### 4.9. Sensory Attributes of Wheat Steamed Bread

Wheat steamed bread was prepared according to the Chinese national standard GB/T 35991-2018 [40]. The sensory attributes and their respective scoring points are specific volume (g/mL, 20 points); width/height ratio (5 points); elasticity (10 points); surface color (10 points); surface structure (10 points); internal structure (20 points); toughness (10 points); viscosity (10 points); and taste (5 points), with a total score of 100. Eight evaluators (five males and three females) aged between 23 and 53 observed, tasted, and evaluated the steamed bread. Each evaluator had a steamed bread sample for each polydextrose addition. The evaluator did not smoke or eat one hour before tasting but could drink water. During the tasting, each evaluator was in a normal physiological state and did not utilize cosmetics or other products with a strong odor. The tasting was carried out one hour before a meal or two hours after a meal.

### 4.10. Scanning Electron Microscopy (SEM)

The wheat bread samples were cooled to room temperature and kept in a −20 °C refrigerator. Before SEM observation, the samples were lyophilized in a freeze dryer. The dried samples were fixed on a sample stage and then sputtered with gold in a vacuum ion particle sprayer (JEC-3000FC, Japan Electronics Co., Ltd., Tokyo, Japan). The sputtering conditions were as follows: working accelerating voltage of 5.0 kV, sputtering current of 30 mA, sputtering time of 130 s, working distance of 10 mm, and sputtering working pressure of 2.0 Pa. The samples were then set on the stage of a scanning electron microscope (JSM-IT 700HR, Japan Electronics Co., Ltd.) and photographed at an accelerating voltage of 25 kV, with 100 to 1200 times magnification. The pressure in the observation chamber was 7.50 × 10^−8^ Pa, with an emission current of 88 μA and a distance of 10 mm between the sample and the lens.

### 4.11. Data Analysis

The data were statistically analyzed using SPSS (Version 17.0, SPSS Incorporated, [45]). For the untreated (CK) and added polydextrose samples, the one-way ANOVA approach and Duncan’s new multiple-range test were used to compare multiple means. Statistical significance was set at the *p* < 0.05 level.

## Figures and Tables

**Table 1:** Effect of adding polydextrose on the texture profiles of steamed bread.

PD-ST Addition (%)	Hardness (g)	Adhesive Force (g)	Adhesiveness (0.1 mJ)	Resilience (0.1)	Cohesiveness (0.1)	Springiness (mm)	Gumminess (g)	Chewiness (mJ)
0	1171 ± 78 ^d^	3.05 ± 0.07 ^a^	0. 2 ± 0.0 ^b^	4.13 ± 0.25 ^ab^	7.80 ± 0.17 ^ab^	10.84 ± 0.11 ^a^	829 ± 35 ^c^	87.7 ± 3.1 ^c^
3	1067 ± 58 ^d^	1.20 ± 1.13 ^b^	0. 2 ± 0.1 ^b^	4.63 ± 0.42 ^a^	8.03 ± 0.15 ^a^	10.89 ± 0.25 ^a^	872 ± 60 ^b^	93.2 ± 7.7 ^bc^
5	1394 ± 71 ^a^	4.17 ± 3.18 ^ab^	3.9 ± 0.3 ^a^	3.97 ± 0.25 ^ab^	7.77 ± 0.21 ^ab^	10.90 ± 0.09 ^a^	1081 ± 86 ^a^	115.7 ± 10.1 ^a^
7	1237 ± 84 ^b^	1.00 ± 0.44 ^b^	3.9 ± 0.2 ^a^	3.97 ± 0.15 ^b^	7.63 ± 0.06 ^b^	10.78 ± 0.14 ^a^	948 ± 59 ^b^	100.3 ± 7.5 ^b^
10	1244 ± 54 ^c^	1.07 ± 0.15 ^b^	4.7 ± 0.7 ^a^	4.01 ± 0.21 ^b^	7.61 ± 0.21 ^b^	10.79 ± 0.11 ^a^	1101 ± 64 ^a^	117.8 ± 9.0 ^a^

Note: All data are expressed as mean ± SD; number of repetitions—*n* = 3. Different superscript letters indicate the significant differences (*p* < 0.05) within the column.

**Table 2:** Effect of added polydextrose on the specific volume of steamed bread.

PD-ST Addition (%)	Volume (cm^3^)	Mass (g)	Specific Volume (cm^3^/g)
0	190.1 ± 0.9 ^b^	95.0 ± 0.5 ^c^	2.00 ± 0.01 ^a^
3	192.1 ± 1.2 ^ab^	99.8 ± 0.9 ^b^	1.92 ± 0.02 ^b^
5	192.5 ± 2.5 ^ab^	100.0 ± 1.3 ^b^	1.92 ± 0.03 ^b^
7	194.4 ± 2.1 ^a^	102.5 ± 0.7 ^a^	1.89 ± 0.02 ^b^
10	195.5 ± 0.6 ^a^	103.4 ± 0.5 ^a^	1.89 ± 0.01 ^b^

Note: All data are expressed as mean ± SD; number of repetitions—*n* = 3. Different superscript letters indicate significant differences (*p* < 0.05) within the column.

**Table 3:** Effect of adding polydextrose on the color of steamed bread.

PD-ST (%)	L	−a	b	ΔE
0	77.39 ± 0.54 ^b^	1.707 ± 0.029 ^a^	12.89 ± 0.25 ^b^	21.03 ± 0.66 ^c^
3	78.40 ± 0.39 ^b^	1.642 ± 0.023 ^b^	13.32 ± 0.17 ^a^	22.34 ± 0.39 ^b^
5	80.44 ± 0.51 ^a^	1.502 ± 0.046 ^c^	13.44 ± 0.28 ^a^	24.35 ± 0.51 ^a^
7	80.15 ± 0.31 ^a^	1.697 ± 0.023 ^a^	13.51 ± 0.12 ^a^	24.09 ± 0.30 ^a^
10	80.33 ± 0.12 ^a^	1.721 ± 0.011 ^a^	13.55 ± 0.11 ^a^	24.39 ± 0.28 ^a^

Note: L indicates the brightness index, a indicates greenish tones, b indicates yellowish tones, and ΔE indicates a color difference. All data are expressed as mean ± SD; number of repetitions—*n* = 3. Different superscript letters indicate significant differences (*p* < 0.05) within the column.

**Table 4:** Effect of added polydextrose on the sensory attributes of steamed bread.

**PD-ST (%)**	**Specific Volume**	**Width/Height Ratio**	**Elasticity**	**Surface Color**	**Surface Structure**
0	8.00 ± 0.00 ^a^	4.00 ± 0.00 ^a^	7.00 ± 0.58 ^a^	7.00 ± 0.58 ^a^	7.40 ± 0.73 ^a^
3	7.00 ± 0.00 ^b^	3.00 ± 0.00 ^b^	7.60 ± 0.45 ^a^	6.80 ± 0.68 ^a^	7.80 ± 0.37 ^a^
5	7.00 ± 0.00 ^b^	3.00 ± 0.00 ^b^	7.20 ± 1.06 ^a^	6.80 ± 0.37 ^a^	8.40 ± 0.45 ^a^
7	6.50 ± 0.00 ^c^	3.00 ± 0.00 ^b^	7.40 ± 0.45 ^a^	6.80 ± 0.89 ^a^	7.60 ± 0.93 ^a^
10	6.50 ± 0.00 ^c^	3.00 ± 0.00 ^b^	6.80 ± 0.37 ^a^	7.40 ± 0.73 ^a^	7.40 ± 0.73 ^a^
**PD-ST (%)**	**Internal Structure**	**Toughness**	**Adhesiveness**	**Taste**	**Total Score**
0	15.40 ± 0.45 ^b^	7.40 ± 0.93 ^a^	7.60 ± 0.93 ^a^	4.60 ± 0.45 ^a^	68.40 ± 2.05 ^a^
3	16.20 ± 0.37 ^ab^	6.80 ± 0.68 ^a^	7.80 ± 0.68 ^a^	4.60 ± 0.45 ^a^	67.60 ± 2.21 ^a^
5	16.20 ± 1.57 ^ab^	7.40 ± 0.45 ^a^	7.60 ± 0.45 ^a^	5.00 ± 0.00 ^a^	68.60 ± 1.59 ^a^
7	16.40 ± 1.48 ^ab^	7.00 ± 0.82 ^a^	8.20 ± 0.37 ^a^	4.80 ± 0.37 ^a^	67.70 ± 2.61 ^a^
10	17.20 ± 1.21 ^a^	7.60 ± 0.93 ^a^	8.40 ± 0.73 ^a^	4.80 ± 0.37 ^a^	69.10 ± 1.69 ^a^

Note: All data are expressed as mean ± SD; number of repetitions—*n* = 8. Different superscript letters indicate the significant differences (*p* < 0.05) within the column.

**Table 5:** Effect of adding polydextrose on the pasting parameters of wheat flours.

PD-ST (%)	Peak Viscosity (cp)	Trough Viscosity (cp)	Breakdown Viscosity (cp)	Final Viscosity (cp)	Setback Viscosity (cp)	Peak Time (min)	Pasting Temp. (°C)
0	2061 ± 15 ^a^	1228 ± 7 ^a^	833 ± 18 a	2522 ± 8 ^a^	1293 ± 14 ^a^	6.11 ± 0.03 ^a^	69.33 ± 0.08 ^d^
3	1854 ± 8 ^b^	1125 ± 15 ^b^	729 ± 10 ^b^	2322 ± 10 ^b^	1197 ± 23 ^b^	6.09 ± 0.08 ^ab^	69.83 ± 0.51 ^cd^
5	1806 ± 6 ^c^	1095 ± 13 ^c^	711 ± 19 ^b^	2285 ± 3 ^c^	1190 ± 13 ^b^	6.09 ± 0.03 ^a^	70.13 ± 0.03 ^c^
7	1634 ± 17 ^d^	1000 ± 28 ^d^	634 ± 18 ^c^	2096 ± 6 ^d^	1096 ± 31 ^c^	6.04 ± 0.10 ^ab^	87.42 ± 0.43 ^b^
10	1481 ± 13 ^e^	913 ± 16 ^e^	569 ± 4 ^d^	1943 ± 10 ^e^	1030 ± 9 ^d^	6.00 ± 0.01 ^b^	87.95 ± 0.09 ^a^

Note: All data are expressed as mean ± SD; number of repetitions—*n* = 3. Different superscript letters indicate significant differences (*p* < 0.05) within the column.

**Table 6:** Effect of adding polydextrose on the thermal properties of wheat flour (0 d).

PD-ST (%)	Δ*H* (J/g)	*T*_p_ (°C)	*T*_o_ (°C)	*T*_c_ (°C)	Peak Width (°C)	Peak Height (0.01 mW/mg)
0	5.57 ± 0.25 ^a^	63.27 ± 0.15 ^d^	57.47 ± 0.06 ^c^	70.00 ± 0.40 ^bc^	5.90 ± 0.10 ^a^	11.77 ± 0.80 ^ab^
3	5.37 ± 0.02 ^a^	63.60 ± 0.17 ^c^	57.70 ± 0.20 ^bc^	70.00 ± 0.20 ^c^	5.80 ± 0.10 ^ab^	11.77 ± 0.30 ^a^
5	5.10 ± 0.11 ^b^	63.97 ± 0.25 ^bc^	57.93 ± 0.15 ^b^	70.30 ± 0.26 ^bc^	5.80 ± 0.01 ^ab^	11.36 ± 0.18 ^a^
7	4.88 ± 0.11 ^bc^	64.67 ± 0.68 ^ab^	58.73 ± 0.21 ^a^	71.00 ± 0.66 ^ab^	5.87 ± 0.15 ^ab^	10.68 ± 0.34 ^b^
10	4.81 ± 0.11 ^c^	64.57 ± 0.15 ^a^	58.77 ± 0.06 ^a^	70.67 ± 0.06 ^a^	5.73 ± 0.06 ^b^	10.84 ± 0.30 ^b^

Note: Δ*H*, Gelatinization enthalpy; *T*_o_, *T*_p_, and *T*_c_ are the onset temperature, peak temperature, and conclusion temperature of gelatinization, respectively. All data are expressed as mean ± SD; number of repetitions—*n* = 3. Different superscript letters indicate significant differences (*p* < 0.05) within the column.

**Table 7:** Effect of adding polydextrose on the thermal properties of retrograded samples (21 d).

PD-ST (%)	Δ*H* (J/g)	*T*_p_ (°C)	*T*_o_ (°C)	*T*_c_ (°C)	Peak Width (°C)	Peak Height (0.01 mW/mg)	Aging (%)
0	1.43 ± 0.19 ^a^	53.80 ± 0.85 ^ab^	46.57 ± 0.21 ^c^	62.53 ± 0.21 ^b^	8.80 ± 0.53 ^a^	2.30 ± 0.28 ^a^	25.72 ± 4.35 ^a^
3	1.05 ± 0.13 ^b^	53.20 ± 3.40 ^ab^	48.20 ± 0.01 ^b^	62.47 ± 0.25 ^bc^	8.50 ± 0.20 ^a^	1.87 ± 0.12 ^b^	19.58 ± 2.51 ^ab^
5	0.82 ± 0.09 ^b^	52.60 ± 0.61 ^b^	49.30 ± 0.10 ^a^	63.73 ± 0.25 ^a^	7.80 ± 0.26 ^b^	1.59 ± 0.14 ^c^	16.19 ± 2.15 ^b^
7	0.82 ± 0.01 ^c^	53.83 ± 0.71 ^ab^	48.27 ± 0.80 ^ab^	61.97 ± 0.25 ^c^	7.90 ± 0.36 ^b^	1.54 ± 0.06 ^c^	16.80 ± 0.40 ^b^
10	0.77 ± 0.04 ^c^	54.50 ± 0.01 ^a^	49.23 ± 0.98 ^a^	62.03 ± 0.25 ^c^	7.60 ± 0.46 ^b^	1.52 ± 0.08 ^c^	16.11 ± 1.10 ^b^

Note: Δ*H*, Gelatinization enthalpy; *T*_o_, *T*_p_, and *T*_c_ are the onset temperature, peak temperature, and conclusion temperature of gelatinization, respectively. All data are expressed as mean ± SD; number of repetitions—*n* = 3. Different superscript letters indicate significant differences (*p* < 0.05) within the column.

**Table 8:** Effect of added polydextrose on the thermo-mechanical parameters of wheat dough without yeast powder.

**PD-ST (%)**	**DDT (min)**	**DST (min)**	**C1–Cs (0.1 Nm)**	**C3 (Nm)**	**C3/C4**
0	3.55 ± 0.36 ^e^	4.83 ± 0.42 ^d^	2.28 ± 0.17 ^a^	1.57 ± 0.02 ^a^	1.149 ± 0.011 ^ab^
3	4.55 ± 0.17 ^d^	7.03 ± 0.32 ^c^	0.74 ± 0.09 ^b^	1.55 ± 0.02 ^a^	1.151 ± 0.014 ^ab^
5	5.32 ± 0.34 ^c^	8.30 ± 0.10 ^a^	0.27 ± 0.07 ^c^	1.46 ± 0.01 ^b^	1.126 ± 0.006 ^c^
7	6.91 ± 1.05 ^b^	7.27 ± 0.78 ^b^	0.05 ± 0.04 ^d^	1.46 ± 0.01 ^b^	1.126 ± 0.014 ^bc^
10	8.59 ± 0.06 ^a^	5.25 ± 0.07 ^d^	0.08 ± 0.02 ^d^	1.32 ± 0.01 ^c^	1.159 ± 0.009 ^a^
**PD-ST (%)**	**C3–C4 (0.1 Nm)**	**C5–C4 (0.1 Nm)**	**α (−0.01 Nm)**	**β (0.01 Nm)**	**γ (−0.01 Nm)**
0	2.05 ± 0.12 ^a^	6.74 ± 0.10 ^a^	4.93 ± 0.31 ^c^	46.73 ± 1.15 ^b^	2.80 ± 1.91 ^a^
3	2.03 ± 0.16 ^ab^	7.05 ± 0.43 ^a^	4.73 ± 0.81 ^bc^	45.27 ± 1.80 ^b^	3.07 ± 1.15 ^a^
5	1.63 ± 0.08 ^d^	6.23 ± 0.15 ^b^	5.47 ± 0.81 ^bc^	45.93 ± 4.69 ^abc^	2.33 ± 1.67 ^a^
7	1.62 ± 0.16 ^cd^	6.04 ± 0.13 ^b^	6.00 ± 0.72 ^ab^	50.47 ± 0.61 ^a^	14.87 ± 1.96 ^a^
10	1.81 ± 0.08 ^bc^	5.11 ± 0.08 ^c^	6.50 ± 0.14 ^a^	41.70 ± 0.99 ^c^	3.10 ± 0.14 ^a^

Note: DDT, Dough development time; DST, dough stability time; C1–Cs, protein weakness; C3, maximum gelatinization torque; C3–C4, starch breakdown; C3/C4, amylase activity; C5–C4, starch setback; α, heating speed; β, gelatinization speed; γ, enzymatic degradation speed. All data are expressed as mean ± SD; number of repetitions—*n* = 3. Different superscript letters indicate significant differences (*p* < 0.05) within the column.

**Table 9:** Effect of polydextrose on starch crystallinity and protein conformation in dough.

**PD-ST (%)**	**R_1022/995_**	**R_1047/1022_**	**R_1068/1022_**	
0	1.0419 ± 0.0006 ^b^	0.9147 ± 0.0022 ^d^	0.7492 ± 0.0038 ^e^	
3	1.0334 ± 0.0004 ^c^	0.9259 ± 0.0019 ^a^	0.7840 ± 0.0049 ^a^	
5	1.0333 ± 0.0015 ^c^	0.9227 ± 0.0007 ^b^	0.7742 ± 0.0023 ^b^	
7	1.0426 ± 0.0004 ^b^	0.9185 ± 0.0009 ^c^	0.7628 ± 0.0012 ^d^	
10	1.0486 ± 0.0017 ^a^	0.9189 ± 0.0002 ^c^	0.7688 ± 0.0001 ^c^	
**PD-ST (%)**	**β-sheet (%)**	**Random coil (%)**	**α-helix (%)**	**β-turn (%)**
0	48.928 ± 0.102 ^d^	17.291 ± 0.043 ^a^	17.281 ± 0.025 ^a^	16.499 ± 0.035 ^a^
3	50.244 ± 0.258 ^b^	16.928 ± 0.073 ^d^	16.807 ± 0.088 ^c^	16.022 ± 0.096 ^c^
5	49.507 ± 0.146 ^c^	17.141 ± 0.041 ^c^	17.073 ± 0.061 ^b^	16.278 ± 0.045 ^b^
7	49.361 ± 0.065 ^c^	17.202 ± 0.014 ^b^	17.145 ± 0.031 ^b^	16.292 ± 0.021 ^b^
10	50.184 ± 0.037 ^a^	16.992 ± 0.015 ^d^	16.826 ± 0.007 ^c^	15.998 ± 0.020 ^c^

Note: PD, Polydextrose; R_1022/995_ and R_1047/1022_ show the degree of short sequences at the surface of starch granules, and R_1068/1022_ shows the interaction between starch and proteins. All data are expressed as mean ± SD; number of repetitions—*n* = 3. Different superscript letters indicate significant differences (*p* < 0.05) within the column.

**Table 10:** Effect of polydextrose on starch crystallinity and protein conformation in steamed bread.

**PD-ST (%)**	**R_1022/995_**	**R_1047/1022_**	**R_1068/1022_**	
0	1.0249 ± 0.0005 ^a^	0.9402 ± 0.0037 ^c^	0.8316 ± 0.0083 ^c^	
3	1.0183 ± 0.0010 ^bc^	0.9473 ± 0.0018 ^b^	0.8554 ± 0.0017 ^b^	
5	1.0175 ± 0.0008 ^c^	0.9501 ± 0.0034 ^b^	0.8644 ± 0.0076 ^b^	
7	1.0201 ± 0.0010 ^b^	0.9351 ± 0.0024 ^c^	0.8306 ± 0.0011 ^c^	
10	1.0162 ± 0.0016 ^c^	0.9565 ± 0.0021 ^a^	0.8784 ± 0.0045 ^a^	
**PD-ST (%)**	**β-sheet (%)**	**Random coil (%)**	**α-helix (%)**	**β-turn (%)**
0	52.587 ± 0.348 ^bc^	16.345 ± 0.081 ^b^	15.853 ± 0.126 ^b^	15.214 ± 0.142 ^bc^
3	52.933 ± 0.235 ^b^	16.283 ± 0.081 ^b^	15.605 ± 0.134 ^bc^	15.178 ± 0.123 ^c^
5	52.456 ± 0.146 ^c^	16.267 ± 0.026 ^b^	15.886 ± 0.179 ^b^	15.391 ± 0.072 ^b^
7	52.669 ± 0.106 ^bc^	16.467 ± 0.014 ^a^	16.169 ± 0.057 ^a^	15.694 ± 0.035 ^a^
10	53.599 ± 0.202 ^a^	16.074 ± 0.064 ^c^	15.489 ± 0.060 ^c^	14.837 ± 0.079 ^d^

Notes: PD, Polydextrose; R_1022/995_ and R_1047/1022_ show the degree of short sequences at the surface of starch granules, and R_1068/1022_ shows the interaction between starch and proteins. All data are expressed as mean ± SD; number of repetitions—*n* = 3. Different superscript letters indicate significant differences (*p* < 0.05) within the column.

## Data Availability

The original contributions presented in this study are included in this article; further inquiries can be directed to the corresponding author.
